# Surgical Treatment of Acute General Peritonitis

**Published:** 1909-09-11

**Authors:** L. A. Bidwell


### Hospital Clinics.

### SURGICAL TREATMENT OF ACUTE GENERAL PERITONITIS.

By L. A. BIDWELL, F.R.C.S.

(A Lecture delivered at the Post-Graduates' College, West London Hospital, on June 22, 1909.)

As you are all aware, disease of the vermiform appendix is by far the most common cause of general peritonitis. Appendicitis is accountable for something like 60 per cent. of such cases, while about 20 per cent. are due to perforations of the alimentary canal, 10 per cent. to diseases of the pelvic organs, and the remainder to various causes, such as rupture of an abscess. I intend to devote the greater part of my lecture then, to cases of acute general peritonitis due to appendicitis, but before doing so will refer to some of the other conditions which produce peritonitis.

Perforations of the alimentary tract most commonly occur in the stomach or duodenum. Perforation of the stomach is most frequently seen in the female and that of the duodenum in the male. Perforations may also occur in the colon; this condition is usually described as due to ulcerative colitis, but it really might be called a perforating ulcer, from the tendency of such an ulcer to perforate. In these cases of perforation of the colon the peritonitis has been usually local rather than general, and in only one case was there what could be called general peritonitis. Two other cases of perforation occurred in ulcers of the sigmoid, forming just above a stricture which was due to malignant disease. When stricture results after operations for the removal of growths from the intestine, the distension of the bowel above the stricture is liable to be followed by ulcers in the wall of the gut, and these ulcers sometimes perforate. In one of these two cases the condition was recognised within six hours of the perforation and an operation was performed, the perforation being closed successfully; in the other case no operation was performed. A typhoid ulcer may also perforate. Here the prognosis is very bad. Such a condition is sometimes difficult to recognise because of its occurrence in delirious patients when, of course, the symptom of pain is absent. I have also seen perforated tuberculous ulcers of the intestine which have produced definite general peritonitis. But the most peculiar case of perforation of the bowel was in a patient who was accidentally shot, the shot perforating the rectum and the sigmoid flexure, producing general peritonitis. A symptom always to be looked for in cases of peritonitis due to perforated gut is the presence of free gas in the peritoneal cavity, as shown by diminution of the liver dulness. This symptom is one which is rather liable to lead to mistakes. If we have great distension of the colon we shall have obliteration or diminution of the liver dulness. It is only when there is no distension of the rest of the abdomen that the symptom is a valuable one. It is present in about two-thirds of the cases of perforation of the stomach and duodenum, also in the perforation of typhoid and tuberculous ulcers, and I have found it present in one case of perforation of the colon; but in the other cases of perforation of the colon and of the sigmoid flexure it was absent. With regard to miscellaneous causes, I have had two or three cases in which general peritonitis has followed perforation or gangrene of the gall-bladder. I have also had a case of general peritonitis caused by rupture of a sub-peritoneal abscess.

I now come to the discussion of symptoms. Pain is practically always present, except in those cases in which perforation occurs during delirium. Usually it is very acute at first, but when a large quantity of pus has formed it becomes much less intense or even disappears entirely. It is remarkable that the patient suffers less pain when the general condition of the peritoneum is in its worst phase. The next symptom is vomiting. This is practically always found, and in a neglected case the vomit is pumped up in large quantities without any effort on the part of the patient. It is bilious in character at first, but afterwards brown-coloured and evil-smelling. It is often described as stercoraceous, but I believe that its misleading appearance is due to partly digested blood. Rather an interesting case came to the West London Hospital about six months ago with a perforation of a duodenal ulcer. There was a history of an old ulcer of the duodenum. The patient had a sudden attack of pain, vomited first some bile-stained fluid, and after about an hour brought up, what his doctor described as, fæcal vomit. When he came into the hospital the right side of the upper abdomen was rigid, and the patient was in most intense pain and was collapsed. With a history of old stomach trouble and of this sudden acute attack, the diagnosis of ruptured duodenal ulcer was made at once. The only point against that diagnosis was the definite assertion of the doctor that there had been fæcal vomiting. If I had believed this I might have changed my diagnosis, but I came to the conclusion that the so-called fæcal vomit was really partially digested blood, the vomit therefore being offensive and dark-coloured. The reason for this altered blood is that when the intestines are congested the mucous membrane becomes œdematous and exudes blood-stained fluid.

A rigor is sometimes noted, but is not at all constant. It is of interest because rigor occurs during the passage of a stone, and in that way we may confuse acute peritonitis with the passage of a gall-stone or renal calculus. The presence of a rigor does not necessarily mean the passage of a stone.

The temperature immediately after perforation is practically always sub-normal; but with the commencement of peritonitis it nearly always rises slightly—a degree or so. Sometimes the temperature is very high, but after a few hours it often falls and remains sub-normal throughout the case.

The pulse is almost without exception rapid and very thin in volume. In abdominal surgery more attention is paid to the condition of the pulse than to the temperature. The aspect of the patient, even at the commencement of the peritonitis, is anxious. His face is drawn, the eyes become sunken, the lips dry and crusted, and we often find a duskiness of the face even after a few hours of peritonitis. Later the " facies hippocratica " becomes evident.

On examining the abdomen the first thing we notice is a limitation of the movements of respiration, which is practically the first sign of a severe peritoneal lesion. In cases of appendix and pelvic trouble causing general peritonitis, the absence of movement will probably be confined to the lower half of the abdomen. The abdomen is not distended at first; but it is nearly always rigid, and in some part of, or over the whole abdomen the muscles are board-like. Later on we find distension and tympanites. We often find shifting dulness on moving the patient about. That dulness does not necessarily mean free fluid in the peritoneal cavity. It is accounted for by parts of the small intestine and of the transverse colon becoming distended with fluid, which shifts as the patient is moved about. Rectal examination causes very much pain and affords little information. There is often pain on micturition. I mentioned that in peritonitis, due to perforation of the gut, free gas is usually found in the peritoneal cavity. But it is very unusual to discover free gas in a general peritonitis due to a perforated appendix. I only remember two cases in which free gas could be detected on percussion, and those were cases in which a large abscess had formed in the pelvis and extended up into the abdomen. They were really cases of localised peritonitis.

In considering the most common cause of general peritonitis—namely, disease of the appendix—we ought first to bear in mind the class of case in which general peritonitis is likely to occur. Let me first draw your attention to the different types of acute inflammation in the appendix. The first is one in which either the whole appendix becomes inflamed and thickened, with the formation of adhesions and flakes of lymph around it, or there may be a stricture in the lumen of the appendix, and a collection of pus or muco-pus in its distal portion. These two forms may subside without any leak or perforation, and the patient recovers. This represents, then, the first type of acute appendicitis. The second type is one in which a minute leak occurs in the appendix, adhesions having previously formed in the neighbourhood of the inflamed part, with the result that the general peritoneal cavity is shut off, and an abscess forms. The third type is much more serious. In this a small leak occurs before any firm adhesions have formed, and only frail adhesions separate the leak in the appendix from the general peritoneal cavity. In such cases we get local spreading peritonitis. The inflammation spreads locally, but does not become absolutely general at first. In the fourth type the appendix becomes gangrenous and ruptures without the preliminary formation of adhesions, producing acute general peritonitis immediately. This latter type, however, is not the only one that is dangerous, for a localised abscess, if neglected, may burst, and produce general peritonitis, or the localised spreading condition may at any moment become general. In my experience the worst cases of general peritonitis are those in which an abscess of considerable size bursts into the general peritoneal cavity. An abscess in connection with the appendix may contain four or five ounces of intensely virulent pus, and when this pus becomes diffused over the peritoneal cavity, the toxins in the pus are rapidly absorbed by the healthy peritoneum. In a case in which a large abscess has burst the patient is dusky-coloured, has a miserable pulse, and looks practically hopeless. The cause of the rapidly fatal termination of acute general peritonitis is undoubtedly toxæmia. The toxæmia arises from the absorption of toxins from the peritoneal cavity, and secondly from the interior of the intestines. That is a point not always recognised. With regard to the absorbent power of the peritoneum, the most rapid and active absorption takes place in the upper part of the abdomen, and the least active from the pelvic portion. I have on several occasions known a patient continue at his work with an abscess, due to appendicitis, in his pelvis, and he has only applied for advice on account of the swelling in the lower part of the abdomen; in fact there have been no urgent symptoms at all. But if the trouble is in the sub-phrenic region the serious symptoms will always occur early. When pus has accumulated in any part of the peritoneal cavity the peritoneum loses its power of absorption.

The next point is the absorption of toxins from the intestines. The Bacillus coli communis exists in large quantities in the lower part of the small and in the large intestine in health, and gives rise to no ill effects; indeed, rather the reverse. But as soon as peritonitis occurs the changes which take place in the walls of the intestine, due to impaired nutrition and to the peritoneal inflammation, evidently cause the secretion by the intestine of a different medium, in which the Bacillus coli communis loses its beneficent character and secretes this highly poisonous toxin. Then, again, the intestines which are subject to acute general peritonitis lose their power of resisting the absorption of toxins, so that any toxin which is poured out in an inflamed piece of intestine will be absorbed very easily and rapidly. Thus the general system is attacked by a double invasion of toxins, the first one coming from the peritoneal cavity, and the second one from the interior of the intestines. Unless we can stop this double invasion the case is likely to be fatal. Nature sometimes endeavours to check the absorption of toxins from the intestinal canal by producing diarrhœa. That is also the aim of those surgeons who treat general peritonitis by repeated saline purges. Modern surgery, however, teaches that the most hopeful way is by laparotomy, and the surgeon proceeds first to remove the cause of peritonitis, and after that to remove all sources of toxic infection. This should be done by thoroughly removing all the pus from the general peritoneal cavity; and in addition the intestinal toxins must be got rid of. This can only be done by emptying those portions of the bowels which are distended with fluid. The fluid with which the bowels are distended is really a virulent culture of Bacillus coli communis. The first indication is not to give any morphia, since this prevents Nature from attempting to get rid of the toxins by diarrhœa. The second indication is to evacuate any pus as soon as possible. In taking a case of general peritonitis following a rupture of the appendix the prognosis will depend almost entirely upon the length of time which has elapsed since the perforation took place: if within six hours the patient will probably be saved. Beyond that time the prognosis becomes graver for every hour of delay. When there is appendix trouble we usually obtain some indication of the nature of the disease by the history of the previous attacks of appendicitis or by the rigidity over the appendix.

A question of some importance is the choice of an anæsthetic. In children suffering from acute septic trouble the giving of chloroform is liable to produce acetonuria, which has an injurious effect upon the child's chances. In the case of a child pure chloroform should not be given; the alternative is ether by the open method, or a mixture of ether and chloroform. But as little chloroform as possible should be given. In adults the question of spinal analgesia should also be considered. When the patient is greatly collapsed, injection of a pint of saline containing an ounce of brandy, per rectum, before the operation is desirable. Intravenous infusion may be given and should be continued during the whole of the operation, as much as five pints being given. I may remind you that saline solution for intravenous injection should have a strength of 1½ drachms to the pint, and not 1 drachm to the pint. The reason of using saline solution of the strength of 1 drachm to the pint is because this represents the composition of Frog's serum, whereas one and a half drachms to the pint more nearly corresponds to the constitution of normal blood serum. Another important point is that the salt itself should be sterilised by boiling. I have often seen saline prepared by carefully boiling the water and then allowing some to cool, afterwards ladling out salt from a dirty box into the cold or lukewarm sterilised water. Such procedure introduces a very great risk to the infusion of saline solution into a vein. It is desirable always to make the saline of double strength, boil the salt with the water, and dilute it by adding an equal quantity of cold sterilised water. The temperature at which the saline is introduced must not exceed 105°. If the saline in the reservoir has a temperature of 105° and the rubber tube through which it is flowing is passed through a basin of fairly warm water just before it enters the vein, any loss of heat which the saline might undergo between the reservoir and the vein is prevented. It is necessary to give one other warning about saline: its introduction must be continuous; we must not stop giving saline and then go on again. If we stop putting in the saline and pinch the rubber tube, a clot is almost certain to form in the vein; if, afterwards, we commence again to inject saline, we shall drive that clot into the right side of the heart. I have seen a patient die suddenly during an operation from that cause.

Coming to the actual operation, the first point is, of course, the position of the incision. If we are dealing with a case of general peritonitis from appendix trouble the incision should be made along the outer border of the right rectus muscle. We should not make the incision too long to start with. With an incision three inches long the nature of the peritonitis can be determined. As soon as we find that we have to deal with an inflamed appendix it is very much better to enlarge the incision than to attempt to remove an inflamed appendix through a small incision. The latter course adds to the liability of tearing the inflamed part or enlarging the rupture. If pus is present the first thing to do is to put a large tube through the incision so as to favour the escape of pus. Having got rid of the first lot of pus, and before flushing out the abdomen, the appendix must be searched for; it is then excised with as much care as possible and its stump closed and invaginated into the cæcum. The next important step is the drainage of the peritoneum. There are three places in which it is necessary to drain. The first of these is the right loin. This is absolutely essential in every case of general peritonitis from appendicitis. The way in which these openings are made is by means of an incision through the skin in the interval between the last rib and the crest of the ilium, behind the posterior axillary line. The incision is made an inch in length. A pair of large forceps is then taken and pushed by a boring motion through the muscles till its point presents under the parietal peritoneum. The finger of the disengaged hand is passed into the wound and feels the forceps beneath the peritoneum. By keeping the finger upon the point of the forceps the operator can ensure that no damage is done to the gut. When the pair of forceps is pushed into the peritoneal cavity its point is made to present into the abdominal wound and a piece of drainage tube is caught by the forceps and dragged across the peritoneal cavity and brought out of the lumbar wound; the portion which is inside the abdomen is directed upwards, the purpose being to drain the right sub-phrenic space. A similar lateral drain is then made on the left side. The tube is introduced in a similar manner, and the left sub-phrenic space is drained.

We have only one other position for drainage, and that is in connection with the pelvis. When pus has collected in the pelvis the best way of draining it is by means of a rectal drain. The only objection to this is the necessity for the operator himself to put one of his fingers into the rectum; it is hardly possible to make use of the finger of an assistant with any assurance. A large pair of forceps is passed into the pelvis and a finger put into the rectum. At a point just above the prostate in a male we can feel the points of forceps through the wall of the rectum, situated about a quarter of an inch from the finger. When the tip of the forceps is felt as close as that to the finger we know that we have got nothing but the wall of the rectum between the forceps and the finger, and we can push the forceps through. We push the rectal wall down towards the anus, bore through the rectum, and the tip of the forceps presents through the anus. A piece of drainage tube is then caught in the open mouth of the forceps which are projecting through the anus, and by means of the forceps the piece of drainage tube is pulled either into Douglas's pouch or the recto-vesical pouch, which it is desired to drain. The tube is left within an inch of the brim of the pelvis, the other end projecting about an inch beyond the anus. The tube is grasped by the rectum and the anus, and is not likely to come away. Thus we have a complete method of drainage—a lateral drain in each loin and the pelvic drain. Having carried out this task, it is now the time to wash out the peritoneum. A great mistake is made in washing out the peritoneum before it has been drained. Only one substance is suitable for this purpose—namely, saline. With antiseptics there is a risk of damaging the peritoneal cells which are already inflamed. Another objection to antiseptics is that a large quantity may be absorbed, and may poison the patient. Having carried out this operation, we close the abdominal wound, leaving a space for drainage and fix the drainage tubes with gut sutures. A point which I have omitted to go into has reference to cases of enormous distension of the small intestine. When the intestines are enormously distended it is quite useless to put them back into the abdomen containing this virulent culture of Bacillus coli communis. We must open the intestines and empty them of their contents. A large loop of intestine is brought outside the abdominal wound, and is packed round with towels. An incision of about an inch long is then made into the convex border of the intestine, the hand, previously washed in saline, is put into the abdomen and an endeavour made to milk out into this opened coil as much as possible of the intestinal contents. In this way one is able usually to empty out the greater part of the contents of the small intestine. The wound in the intestine is then sewn up and the coil replaced. Some surgeons have recommended putting in saline solutions after removing the contents, thereby avoiding shock.

After-treatment is important. The first thing to be considered is the position of the patient. When the patient is suffering from intense shock he has to be lying low, but after the first shock has subsided he may be put in Fowler's position—half sitting up—for the purpose of preventing any pus left in the peritoneum from tracking up to the sub-phrenic spaces. What we want to do is to make it track towards the pelvis, where it will be immediately evacuated by the rectal drainage. Stimulation is most important, and it is hardly possible to give too much in the way of stimulants. As soon as the patient can swallow he should be given brandy or champagne. When the patient has got back to bed he should be given a hypodermic injection of strychnine—one-thirtieth of a grain—and half this dose should be repeated every four hours. I do not like the administration of strychnine during the operation. The next thing is the administration of salines. The salines are given half a pint at a time every four hours. This is much better than the common plan of giving a pint every six hours. I add to each pint of saline injection a tablespoonful of grape sugar. The sugar can be absorbed from the rectum just as easily as salt, and it is one of the most important substances in influencing the nutrition of the body. One point upon which I insist is that the patient must be given three pints of fluid per day. If only one pint can be taken by the mouth the other two pints must be given by the rectum. The total amount taken per day must in any case be made up to three pints. Sometimes we cannot introduce the saline injection into the rectum owing to the rectal drainage. As a rule, the rectal drainage does not interfere with the saline injection, but in some cases it does, and in those cases we must give salines subcutaneously. The saline should be introduced beneath the pectoral muscles or at the side of the abdomen. It is not a pleasant way, and one has to be careful in subcutaneous injections, not only about the sterilisation of the saline itself, but also about the sterilisation of the patient's skin. With regard to the management of the bowels, to encourage the passage of flatus a rectal tube is passed up and left *in situ* five minutes before each saline is given. On the evening of the second day I nearly always give two grains of calomel, which very seldom gives rise to any discomfort. On the following morning usually a simple enema is given. In most cases this is all that is necessary. If, however, it fails, I give a magnesium sulphate injection, made by dissolving an ounce of Epsom salts and half a drachm of turpentine in a pint of gruel. That is injected as high up as it will go, and it nearly always produces some result. Another method of assisting the calomel is by giving a mixture containing two drachms of mag. sulph., 5 minims of tincture of belladonna, and 10 minims of tincture of nux vomica every four hours. As to intestinal antiseptics, the only one which I should feel inclined to use is a culture of lactic acid bacilli. The most scientific way of attacking the Bacillus coli communis is to put in its adversary, the lactic acid bacillus, and let them fight it out.

With regard to food, one of my fads is that milk should not be given until the bowels have acted. I object to milk because it produces a bulky motion, and is certainly constipating. What the patient requires is undoubtedly sugar and water. The simplest way of giving sugar and water is by means of raisin tea. It is made by pouring boiling water upon raisins, and pouring off the fluid afterwards. The fluid contains grape sugar and also has the pleasant muscatel flavour of the raisin. Besides that, we give barley water and albumin water and occasionally beef-tea. Personally I do not believe much in the nutritive value of beef-tea. It is a stimulant rather than a nutriment, and like the meat extracts it does no harm and may do good. But the staple things are barley water, albumin water, and raisin tea. Directly the vomiting has ceased and the bowels have acted the diet can be enlarged by giving junket and custards, and on the fourth day fish or chicken can be given. A question may be asked as to how long these drainage tubes should remain in position. The rectal drainage tube should remain for two days. It is seldom necessary to leave the other two drainage tubes for more than three or four days. A case of general peritonitis that is going to get well progresses very rapidly, and after the first twenty-four hours, if no more toxins are absorbed, the prognosis is good.

